# Why We Do Need Another Medical Journal…

**DOI:** 10.3390/healthcare1010001

**Published:** 2013-01-30

**Authors:** Manu L. N. G. Malbrain

**Affiliations:** *ICU and High Care Burn Unit Director*, Ziekenhuis Netwerk Antwerpen, ZNA Stuivenberg, Lange Beeldekensstraat 267, B-2060 Antwerpen 6, Belgium; E-Mail: manu.malbrain@skynet.be; Tel.: +32-3-217-7399; Fax: +32-3-217-7574

The English word “health” comes from the Old English word hale, meaning “wholeness, a state of being and feeling whole, sound or well” [1]. Hale comes from the Proto-Indo-European root “kailo”, meaning “whole, uninjured, of good omen”. Kailo comes from the Proto-Germanic root “khalbas”, meaning “something (un)divided”. The Medilexicon’s medical dictionary defines health as “The state of the organism when it functions optimally without evidence of disease or abnormality” [2]. The World Health Organisation (WHO) defines health as a state of complete physical, mental and social well-being and not merely the absence of disease or infirmity [3]. Although this definition is controversial and especially the wording “complete” is open for subjective interpretation and bias, it is the most widely used and accepted. “We must turn to nature itself, to the observations of the body in health and in disease to learn the truth”, as was nicely stated by Hippocrates. While Voltaire took it one step further: “The art of medicine consists of amusing the patient while nature cures the disease”. A disease state can then be defined as an impairment of normal physiological function affecting all or part of an organism, producing characteristic symptoms; illness or sickness in general. In practice, other definitions and the International Classification of Disease (ICD) are more commonly used to define and quantify the different components of health and/or the absence of it.

The English word “care” comes from the Old English word cearu (n), cearian (vb), of Germanic origin; related to the Old High German chara, meaning “to lament”, and the Latin garrīre, meaning “to gossip”. Healthcare hence can be defined as the prevention, treatment (cure), and management (care) of illness and the preservation of mental and physical well-being through the services offered by the medical and allied health professions. According to the Institute of Medicine the care given should be safe, avoiding iatrogenicity and preventable injuries (as was nicely pointed out by Alexander the Great: “I am dying with the help of too many physicians.”); effective and efficient, based on scientific knowledge and clinical guidelines; patient centered; fast and timely, reducing wait times; and consistent regardless of patient characteristics, socio-economic status and demographics [4]. There are two aspects to healthcare, physical and mental health. According to the WHO, mental healthcare is “a state of well-being in which every individual realizes his or her own potential, can cope with the normal stresses of life, can work productively and fruitfully, and is able to make a contribution to her or his community.” Regardless of the definition used, assessment of mental health remains subjective.

However, to play the devil’s advocate, a recent editorial published in The Lancet stated that health is not a “state of complete physical, mental, and social well-being”, as enshrined in the WHO’s famous founding constitution, nor is it “merely the absence of disease or infirmity” [5]. The WHO formulation was supposed to provide a transformative vision of “health for all”, one that went beyond the prevailing negative conception of health based on an “absence” of pathology. However, the above-mentioned definitions will not “survive” in an era marked by new understandings of disease at molecular, individual, and societal levels. Given that we now know the important influence of the genome in disease, even the most optimistic health advocate surely has to accept the impossibility of risk-free well-being [5].

If you are reading this editorial, you are “looking” at the first issue of *Healthcare* (ISSN 2227-9032), yet another international medical journal you may think. Is this really the case? While there is an exponential increase in open access and scholarly journals over the last years, the main focus goes to very dedicated specialty journals with high impact factor. The result is that good quality journals are receiving increasing numbers of submissions, resulting in slow processing times for reviewing and publication and a “gamble” in which perfectly sound papers can eventually be rejected [6]. Let me give you 10 simple but good reasons why you should consider submitting your next original articles, critical reviews, editorials, research notes and short communications to *Healthcare*. I must admit that I always ask the question “give me 10 good reasons why I should use your drug” to a new sales representative visiting me with yet another super-drug, and you will be amazed that many hit the road, ill-prepared, stuttering after they explained and documented the second advantage of the new drug compared to your standard of care. First, as explained above, in the crowded market of dedicated journals there is a place for a journal with a very broad scope, which publishes original theoretical and empirical work, covering all aspects in the interdisciplinary area of healthcare research. Second, *Healthcare* is a peer-reviewed journal, all manuscripts are evaluated by independent anonymous referees in the usual way, but the editors will ensure that this is done as speedily as possible. Third, it is an open access journal managed by a professional office in Switzerland, as such it is free for readers and readily available on the Internet, accounting for a high availability and high visibility. The true advantage of Open Access is that virtually everyone with internet access (and not only people working at institutions that can afford the subscription fees) can download and read the articles at no cost. This increases the number of people who read and cite the work [6]. Moreover, and fourth, it is also free for authors submitting their work during the next two years (after that period a small article processing fee or charge may apply). Fifth, *Healthcare* also offers the possibility to provide help for non-native English speaking authors for those articles that need major editing and formatting and/or English editing. Sixth, there is no restriction on the word length of the papers as we encourage researchers to publish their experimental and theoretical results in as much detail as possible. For theoretical papers, full details of proofs must be provided so that the results can be checked; for experimental papers, full experimental details must be provided so that the results can be reproduced. Additionally, electronic files or software regarding the full details of the calculations, experimental procedure, *etc*., can be deposited along with the publication as “Supplementary Material”. Seventh, *Healthcare* is a member of the Committee on Publication Ethics (COPE). The Multidisciplinary Digital Publishing Institute (MDPI) takes the responsibility to enforce a rigorous peer-review together with strict ethical policies and standards to ensure to add high quality scientific works to the field of scholarly publication. Several open access journals published by MDPI (such as Sensors, Molecules, Marine Drugs, and Viruses to name a few amongst others), have been indexed by PubMed and/or SCI. Eight, *Healthcare* ensures rapid publication and accepted papers are immediately published online. Once a manuscript is accepted, it can be prepared for on-line publication much faster than for a print journal. Furthermore, there is no need to wait until an “issue” is complete, so each article will be published as soon as it is ready. The result is that work gets published faster and cited sooner [6]. Ninth, the advisory and editorial board of *Healthcare* is a multicultural, multilingual, international mixture of physicians, engineers, managers, and other health care related professionals, all being experts in their field. Finally, *Healthcare* supports the set up of special issues focusing on hot topics related to a special and specific research field. Although these will often be edited by a member of the Editorial Board, we welcome proposals from the whole healthcare community. For example, a Special Issue may be devoted to selected papers presented at a relevant conference. If you are not yet convinced, I even could add an eleventh reason related to the fact that the management of *Healthcare* is based in Switzerland of all places: indeed, in the perfect world, the police are British, the cooks French, the engineers German, the lovers Italian, and it is all very well organized and run by the Swiss. In case you may wonder or would have forgot, hell is a place where the police are German, the cooks British, the engineers French, the lovers Swiss, and it is all organized and run by the Italians…

*Healthcare* wants to foster education and to promote any research on healthcare in its most broadest sense: physical health related topics like clinical research and nursing care research/diagnosis, treatment, and prevention of disease; chronic illnesses (diabetes and heart failure, obesity, arterial hypertension, asthma and chronic obstructive lung disease); patient and hospital care (women’s health with obstetrics and gynecology, contraception and reproductive health, infection control and wound care, pediatric and neonates care, severe burns, various surgery, patient safety); electronic health records and online portfolio based learning (use of computers, simulations, virtual reality, epidemiologic modeling, molecular, pharmacologic, and disease modeling); occupational therapy in healthcare (disability and rehabilitation, brain injury sequelae); home and hospice health care; critical care and emergency healthcare; exercise nutrition and health (hygiene, athletic training and sports); long-term healthcare; general healthcare (quality, materials, information technology, economics, social, ethical and political context of health and health policy implications, education, health services); physical therapy and last but not least mental healthcare.

The path ahead is clear but only time will tell whether *Healthcare* will follow this path and become a successful peer-reviewed, open access journal amongst its peers. The success will mainly be dependent on whether *Healthcare* is PubMed indexed in the future, and therein lies the real challenge. With your help and support this is just a small step ahead… so why not submit your next paper to *Healthcare*, and remember for the next two years it is completely free of charge…
